# Raising Giants

**Published:** 1873-10

**Authors:** 


### RAISING GIANTS.

King Frederick William, of Prussia, father of Frederick the Great, determined to raise to order soldiers whose stature should meet his views of what grenadiers to serve royalty should be. The army was his hobby, and tall men his special admiration. He had a regiment at Potsdam that was the talk of the world, on account of their heads and shoulders being far above ordinary humanity. There were three battalions of 800 each, 2400 in all, perfect Anaks, the shortest of the men being seven feet, and the tallest nine. Such lofty beings were procured from all countries in Europe, without regard to cost. James Kirkman, an Irish recruit, could not be had until $6000 were paid. Tall men were decoyed and put into the service at all hazards. Next he compelled them to marry unusually tall women, whether they consented or not. Prussia is rich in very tall subjects, the descendants of those gigantic grenadiers; these are far taller than the full-blooded Kentuckians. In spite of his eccentric majesty's efforts, however, Nature would have her own way, and the children of such parentage were not all tall at maturity. Then again, another law came into operation to thwart the monarch's ambition to develop a race of monster men. Short men very generally prefer tall wives, and tall women, dapper little husbands. Of course there is no very philosophical way of accounting for taste, but such is the fact. There is a growth limitation to plants and animals. On reaching the predestined dimensions, those active artisans that built up the body, as far as the law of limitation requires, cease laboring, and a permanent type of size is thus established. It is impossible to go counter to those laws and raise giants of any kind. A few individuals, transcending their kindred in altitude, are apparently accidental, or, at least, are beyond explanation; but anomalies in that respect, like monstrosities, cannot be perpetuated through generations.—*Med. & Sur. Rep.*

